# Physical activity during adolescence and the development of cam morphology: a cross-sectional cohort study of 210 individuals

**DOI:** 10.1136/bjsports-2017-097626

**Published:** 2017-08-10

**Authors:** Antony Palmer, Scott Fernquest, Mo Gimpel, Richard Birchall, Andrew Judge, John Broomfield, Julia Newton, Mark Wotherspoon, Andrew Carr, Sion Glyn-Jones

**Affiliations:** 1 Nuffield Department of Orthopaedics, Rheumatology, and Musculoskeletal Sciences, University of Oxford, Oxford, UK; 2 Southampton Football Club, Southampton, UK; 3 MRC Lifecourse Epidemiology Unit, University of Southampton, Southampton, UK

**Keywords:** physical activity, hip, football, MRI, sport

## Abstract

**Introduction:**

Cam morphology is a strong risk factor for the development of hip pain and osteoarthritis. It is increasingly thought to develop in association with intense physical activity during youth; however, the aetiology remains uncertain. The study aim was to characterise the effect of physical activity on morphological hip development during adolescence.

**Methods:**

Cross-sectional study of individuals aged 9–18 years recruited from Southampton Football Club Academy (103 male) with an age-matched control population (52 males and 55 females). Assessments included questionnaires and 3 Tesla MRI of both hips. Alpha angle, epiphyseal extension and epiphyseal tilt were measured on radial images.

**Results:**

Alpha angle and epiphyseal extension increased most rapidly between ages 12 and 14 years. Soft-tissue hypertrophy at the femoral head-neck junction preceded osseous cam morphology and was first evident at age 10 years. The greatest increase and highest absolute values of alpha angle and epiphyseal extension were colocalised at 1 o’clock. Maximum alpha angles were 6.7 degrees greater in males than females (p=0.005). Compared with individuals who play no regular sport, alpha angles were 4.0 degrees higher in individuals who play sport for a school or club (p=0.041) and 7.7 degrees higher in individuals competing at a national or international level (p=0.035). There was no association with leg dominance.

**Conclusions:**

Sporting activity during adolescence is strongly associated with the development of cam morphology secondary to epiphyseal hypertrophy and extension with a dose-response relationship. Males participating in competitive sport are at particularly elevated risk of developing cam morphology and secondary hip pathology.

## Introduction

Cam morphology of the hip is a strong risk factor for the development of hip pain and osteoarthritis.^1^ At present, the pathogenesis of cam morphology remains poorly understood.^2^ The development of interventions to prevent cam formation and hence secondary injury and osteoarthritis requires an improved understanding of its causation.

Studies to date conclude that cam morphology develops around the time of physeal closure^3–7^; however, it is not clear when morphological changes are first evident. This is critical to the timing of possible preventative interventions. The prevalence of cam morphology is higher in adult athlete cohorts compared with non-athletic cohorts,^8^ which may explain the increased rates of hip osteoarthritis in retired footballers.^9–11^ Small studies suggest a higher prevalence of cam morphology among athletes for a number of sports including soccer,^12^ basketball^4^ and ice hockey.^13^ One proposed mechanism of cam formation is epiphyseal extension at the anterosuperior head-neck junction.^14^ However, studies demonstrate limited correlation between epiphyseal extension and subsequent cam morphology.^7 15^ These studies address bony morphology and not the cartilaginous structures of skeletally immature hips. The lower prevalence of cam morphology among females compared with males suggests a possible gender-specific pathogenesis.^16^

Aims of this study were to i) explore when cam morphology first develops, ii) characterise the mechanism of cam development and iii) identify associations with cam morphology.

## Methods

### Study design

Cross-sectional cohort study (participants will subsequently be followed-up longitudinally).

### Population

Individuals aged 9–18 years were recruited from Southampton Football Club (SFC) Academy and local schools (controls) (see online supplementary figure S1for recruitment flowchart and supplementary data for power calculations). Cohorts were loaded for younger age groups to ensure an adequate number of individuals with an open physis and to compensate for loss to follow-up during the planned longitudinal study.

10.1136/bjsports-2017-097626.supp1 Supplementary file 1

10.1136/bjsports-2017-097626.supp2 Supplementary file 2

At SFC, randomly selected individuals from each age category were invited to participate (two individuals declined). The control population was recruited through public engagement events at local schools. Specific age groups were targeted to recruit approximately age-matched cohorts, but no individual was declined participation. The only exclusion criterion was previous hip surgery.

### Assessment

#### Participant demographics and activity levels

Questionnaires were completed with the assistance of parents where appropriate and included the collection of patient demographics (age, gender, body mass index (BMI), leg dominance) and activity levels. Activity was evaluated using three methods: i) SFC Academy versus general population controls; ii) three-tier classification denoted ‘Activity Level’: a) ‘no sport’: no regular sport besides physical education classes at school, b) ‘sport’: regularly play sport for school or club team, c) ‘athlete’: compete at national or international level; iii) Physical Activity Questionnaire (PAQ) for older children (aged 9–13 years) and adolescents (aged 14–18 years).^17^ This collects information on sport and exercise undertaken during an average week and provides a summary score of physical activity levels (a score of 1 indicates low physical activity, whereas a score of 5 indicates high physical activity). It differentiates between active and inactive individuals but does not measure duration, frequency or intensity of activity.^18^

##### Imaging

Cam morphology was assessed with MRI of both hips using a 3 Tesla Philips Achieva platform and torso coil (Philips Healthcare). Two morphological sequences were acquired: three-dimensional (3D) water selective fluid (WATSf) to image joint cartilage and bone, and 3D proton density fat saturation (PDFS) to image the physeal scar (see online supplementary data for sequence parameters).

3D multiplanar reconstructions were performed using OsiriX Software (V.6.0.2, Pixmeo). Radial images were acquired around the axis of the femoral neck at 30 degree intervals (see online supplementary figure S2).

10.1136/bjsports-2017-097626.supp4 Supplementary file 4

### Imaging outcome measures

**Figure 1:**
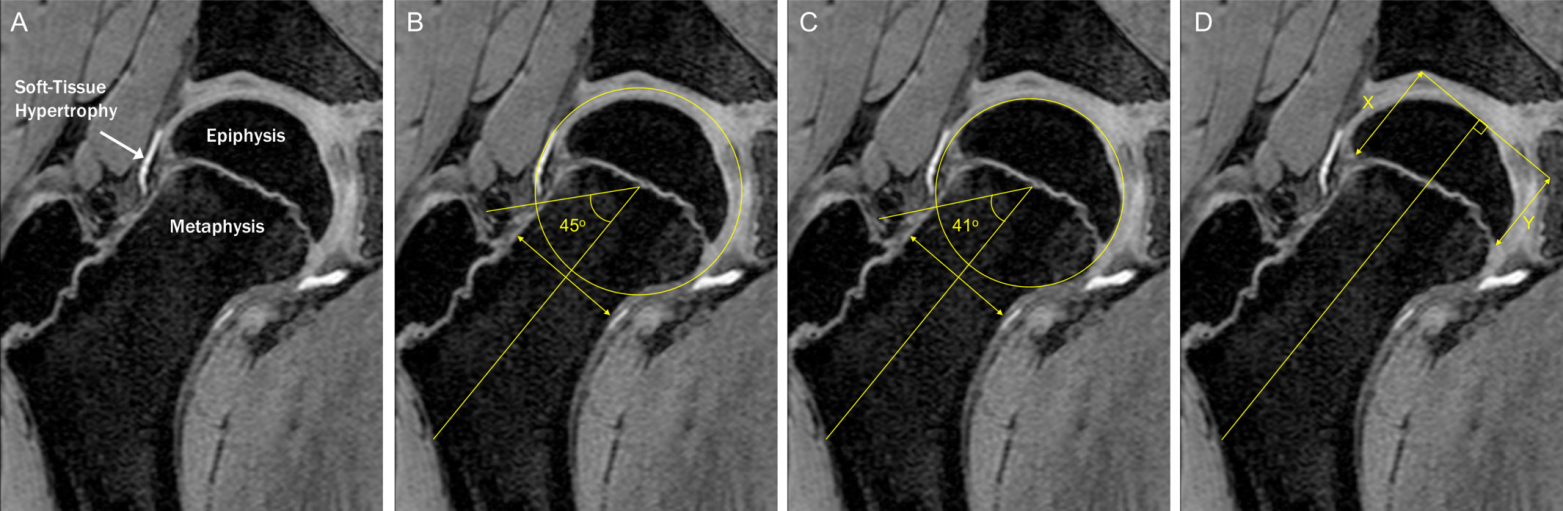
Soft tissue hypertrophy at the head-neck junction preceded epiphyseal extension and osseous cam morphology (**A**). Alpha angle was calculated by drawing a line from the centre of a best-fit circle surrounding the femoral head to the midpoint of a line transecting the narrowest portion of the femoral neck. A further line was then drawn from the centre of the best-fit circle to where the contour of the femoral head first exits this circle. The alpha angle is the angle between these two lines and was measured for cartilage (**B**) and bone (**C**). Epiphyseal extension (**D**) was quantified by measuring the distance from the medial femoral head to the most distal extent of the epiphysis along a line parallel to the axis of the femoral neck as was created when measuring the alpha angle. This distance was then divided by the diameter of the femoral head to produce a standardised ratio. Epiphyseal tilt was assessed as the ratio between epiphyseal extension on opposing sides of the femoral head (X/Y).

Cam morphology was quantified using the alpha angle for bone and cartilage, which was treated as a continuous variable given there is no agreed diagnostic threshold (figure 1).^19^ Radiographic epidemiological studies suggest alpha angles above 60 degrees are elevated and potentially diagnostic.^20^ Cartilage alpha angle was chosen as the primary outcome measure because in the skeletally immature hip the secondary ossification centre does not accurately reflect overall hip shape. Furthermore, it is non-ossified structures that impact in femoroacetabular impingement. The physis of each hip was scored as ‘open’ or ‘closed’ (see online supplementary figure S3).

10.1136/bjsports-2017-097626.supp5 Supplementary file 5

Epiphyseal morphology was quantified using epiphyseal extension, which measures the distance the epiphysis extends along the femoral neck expressed as a ratio of femoral head diameter. Physeal tilt was quantified as the ratio of epiphyseal extension on either side of the physis (figure 1).^13^

Alpha angle and epiphyseal extension were measured using custom-developed software on the radial slices at 11 o’clock, 12 o’clock, 1 o’clock, 2 o’clock and 3 o’clock (figure 1) (see online supplementary data for reproducibility results). These positions were selected as they include the most frequent locations of cam morphology and pilot data suggested the magnitude of cam morphology was greatest at 1 o’clock.^21^ To account for variation in the location of cam morphology, the primary outcome measure was maximum cartilage alpha angle from 11 o’clock through to 3 o’clock.

Measurements were performed by an academic orthopaedic clinician (AP) blinded to participant information. Reproducibility readings were performed by a further academic orthopaedic clinician (SF).

### Statistical analysis

Statistical calculations were performed using STATA V.14.1 (College Station, Texas, USA). Distribution of values was examined using histograms and kernel density plots. Comparison of means was undertaken using an independent two-tailed Student’s t-test for parametric data and Wilcoxon rank sum test for non-parametric data. Linear regression modelling was adopted to assess variables that predict alpha angle and epiphyseal extension. Stepwise estimation identified variables for inclusion in multivariate analysis as age, gender and activity level. Residual plots of the regression models demonstrated normal distributions but evidence of heteroscedasticity. Therefore, the Huber-White-Sandwich estimator was adopted with clustering for laterality to account for the inclusion of left and right hips that are not independent measurements. Interactions were evaluated with linear regression of each combination of variables that predict maximum alpha angle and epiphyseal extension. None reached statistical significance, hence no interaction terms were included in the multivariate models. Statistical significance was set at p<0.05. MRI outcomes were incomplete due to claustrophobia or movement artefact in five hips (two participants in SFC cohort and one female control) and the remaining dataset was complete. No imputation was performed.

## Results

### Participant demographics

The study cohort consisted of 103 males from SFC (mean age 152 months), 52 control males (mean age 153 months) and 55 control females (mean age 167 months) (see online supplementary table S1). Mean BMI was 18.5 (SD 2.6) in the SFC cohort, 19.5 (SD 3.2) in control males and 20.4 (SD 3.7) in control females.

10.1136/bjsports-2017-097626.supp3 Supplementary file 3

In the SFC cohort, the dominant leg was right in 77 individuals, left in 20 individuals, with 6 ambipedal. In male controls, the dominant leg was right in 36 individuals, left in 9 individuals, with 7 ambipedal. In female controls, the right leg was dominant in 41 individuals, left in 6 individuals, with 8 ambipedal. The primary sport of male and female active controls was football or rugby in 31 individuals and non-kicking sports in 40 individuals.

### Activity scores

There were three classification systems for measuring activity: i) Participants were classed as SFC cohort (n=103), control males (n=52) or control females (n=55). ii) Participants were classed as ‘no sport’, ‘sport’ or ‘athlete’. All SFC participants were classed ‘athlete’. Control males consisted of 13 ‘no sport’ and 39 ‘sport’. Control females consisted of 22 ‘no sport’, 31 ‘sport’ and 2 ‘athlete’. iii) Participants completed the PAQ. Mean PAQ for males classified as ‘athlete’ was 3.08 (SD 0.73), ‘sport’ was 2.92 (SD 0.70) and ‘no sport’ was 2.66 (SD 0.67). Mean PAQ for females classed as ‘athlete’ was 3.11 (SD 0.59), ‘sport’ was 2.62 (SD 0.63) and ‘no sport’ was 2.07 (SD 0.63). Mean PAQ decreased with age in all groups.

### Quantitative MRI morphology

#### Alpha angle

**Figure 2:**
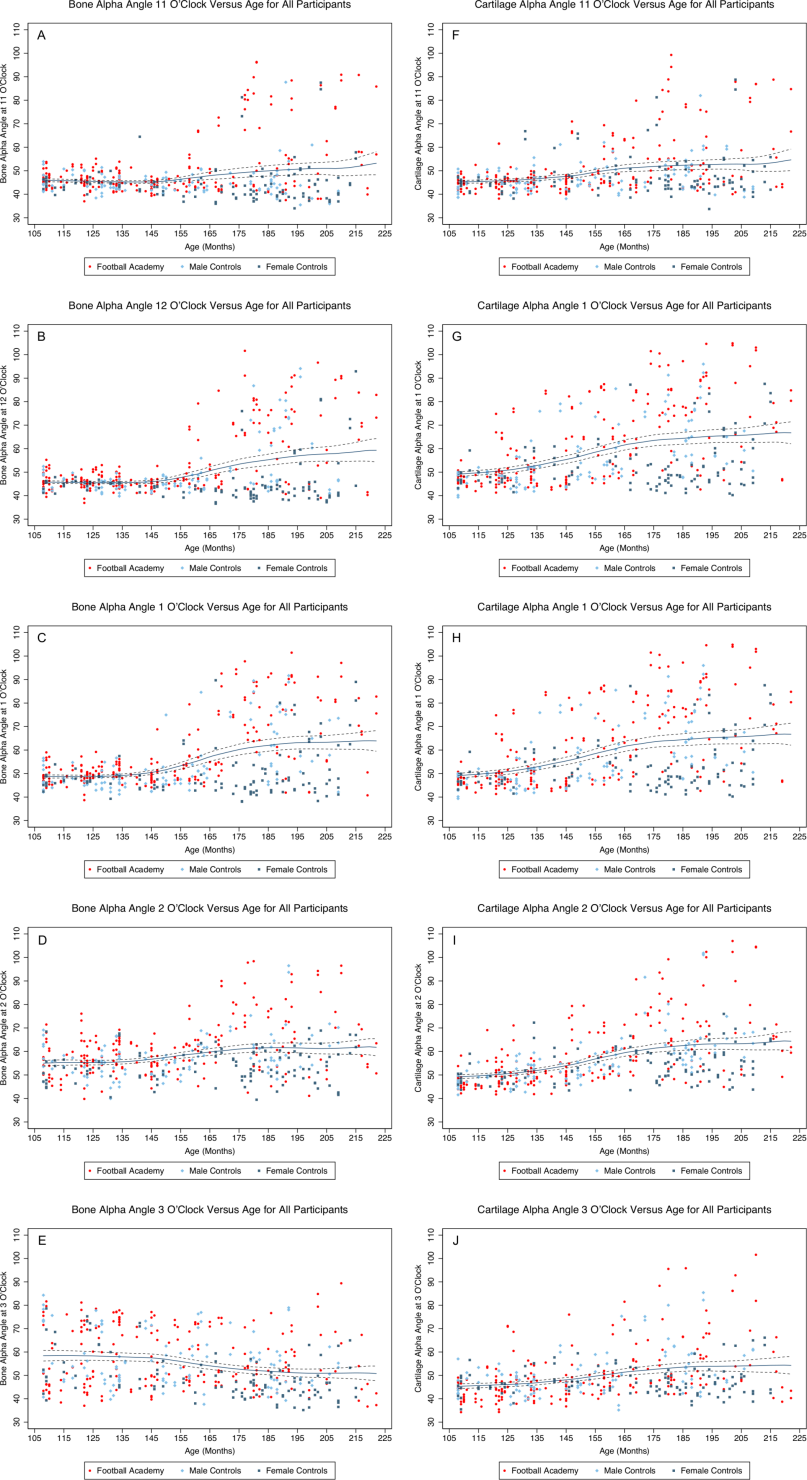
Bone (A–E) and cartilage (F–J) alpha angle vs age in all participants at 11 o’clock (A and F), 12 o’clock (B and G), 1 o’clock (C and H), 2 o’clock (D and I) and 3 o’clock (E and J) with polynomial regression fit and 95% CI.

Cartilage alpha angle was greatest at the 1 o’clock position before and after physeal closure, and while values increased at all positions with age, the greatest increase was also at 1 o’clock (table 1). The most rapid increase in alpha angle was between age 12 and 14 years, with only a small increase beyond 14 years (table 2). Contrary to cartilage alpha angles, bone alpha angles were never elevated at the 1 o’clock position before age 12 years (see figure 2 and online supplementary figure S7).

**Table 1:** Mean cartilage alpha angle, mean epiphyseal extension and mean epiphyseal tilt with an open and closed physis for all participants

Mean cartilage alpha angle						
Clockface position	Open physis (247 hips)		Closed physis (168 hips)		t-Test	
	Mean	SD	Mean	SD	Difference	p Value
11 o’clock	46.69	6.23	53.15	13.67	+6.46	<0.001
12 o’clock	49.99	9.10	60.29	15.39	+10.30	<0.001
1 o’clock	53.18	11.20	65.23	17.21	+12.06	<0.001
2 o’clock	52.21	7.78	63.14	13.94	+10.93	<0.001
3 o’clock	47.24	7.78	53.45	12.60	+6.21	<0.001
Maximum all positions	57.56	11.67	70.80	16.04	+13.24	<0.001

Mean physeal extension						
Clockface position	Open physis (247 hips)		Closed physis (168 hips)		t-Test	
	Mean	SD	Mean	SD	Difference	p Value
11 o’clock	0.716	0.037	0.770	0.052	+0.055	<0.001
12 o’clock	0.673	0.042	0.767	0.070	+0.095	<0.001
1 o’clock	0.682	0.052	0.799	0.075	+0.117	<0.001
2 o’clock	0.655	0.054	0.784	0.065	+0.130	<0.001
3 o’clock	0.577	0.051	0.690	0.063	+0.113	<0.001

Mean physeal tilt						
Clockface position	Open physis (247 hips)		Closed physis (168 hips)		t-Test	
	Mean	SD	Mean	SD	Difference	p Value
11 o’clock	1.59	0.180	1.40	0.153	−0.193	<0.001
12 o’clock	1.52	0.169	1.44	0.169	−0.089	<0.001
1 o’clock	1.56	0.220	1.58	0.220	+0.024	0.270
2 o’clock	1.31	0.199	1.42	0.207	+0.107	<0.001
3 o’clock	0.93	0.100	1.05	0.126	+0.120	<0.001

**Table 2:** Predictors of maximum cartilage alpha angle

		Number of hips	Univariate regression			Multivariable regression 1*			Multivariable regression 2†			Multivariable regression 3‡		
			Coefficient	95% CI	p Value	Coefficient	95% CI	p Value	Coefficient	95% CI	p Value	Coefficient	95% CI	p Value
Months of age		420	0.242 –	0.14 to 0.34	0.020	0.268	0.17 to 0.37	0.019	0.273	0.18 to 0.37	0.018	0.290	0.10 to 0.480	0.033
Age category (years)	9–10	118	–	–	–									
	11–12	92	6.08	5.94 to 6.21	0.001									
	13–14	80	16.23	7.42 to 25.03	0.027									
	15–16	100	17.97	9.37 to 26.56	0.024									
	17–18	30	18.48	13.67 to 23.29	0.013									
Physis	Open	247	–	–	–									
	Closed	168	13.24	6.73 to 19.74	0.025									
Gender	Male	310	–	–	–	–	–	–				–	–	–
	Female	110	−6.05	−9.74 to 2.35	0.031	−6.66	−7.26 to −6.06	0.005				−9.64	−14.75 to −4.53	0.027
BMI		420	1.40	1.02 to 1.78	0.013									
Dominant leg	Dominant	231	–		–									
	Non-dominant	189	0.856	−38.21 to 39.93	0.827									
Cohort	Male control	104	–	–	–				–	–	–			
	Female control	110	−3.37	−4.22 – −2.52	0.013				−7.42	−8.01 to −6.83	0.004			
	Football academy	206	4.05	−0.36 to 8.46	0.054				4.33	0.67 to 7.99	0.042			
Activity level	No sport	70	–	–	–	–	–	–						
	Sport	140	6.21	3.37 to 9.05	0.023	4.03	0.74 to 7.33	0.041						
	Athlete	210	10.50	4.38 to 16.61	0.029	7.63	2.23 to 13.04	0.035						
Physical Activity Questionnaire		420	−3.15	−5.58 – −0.71	0.039							1.52	−6.48 to 9.53	0.250
Table 2b Predictors of epiphyseal extension at 1 o’clock														
Months of age		420	0.0018	0.0018 to 0.0019	<0.001	0.0019	0.0024 to 0.0317	0.002	0.0019	0.0018 to 0.0020	0.002	0.0019	0.0017 to 0.0021	0.006
Age category (years)	9–10	118	–	–	–									
	11–12	92	0.0384	0.0276 to 0.0492	0.014									
	13–14	80	0.0929	0.0848 to 0.1011	0.004									
	15–16	100	0.1375	0.0804 to 0.1945	0.021									
	17–18	30	0.1571	0.0840 to 0.2301	0.023									
Physis	Open	247	–	–	–									
	Closed	168	0.1169	0.1006 to 0.1331	0.007									
Gender	Male	310	–	–	–	–	–	–				–	–	–
	Female	110	0.0085	−0.0064 to 0.0234	0.087	−0.0058	−0.0203 to −0.0087	0.124				−0.0214	−0.0506 to 0.0077	0.068
BMI		420	0.0127	0.0103 to 0.0150	0.009									
Dominant leg	Dominant	231	–	–	–									
	Non-dominant	189	0.0047	−0.1828 to 0.1923	0.802									
Cohort	Male control	104	–	–	–				–	–	–			
	Female control	110	0.0221	0.0191 to 0.0252	0.013				−0.0062	−0.0105 to −0.0029	0.034			
	Football academy	206	0.0205	0.0030 to 0.0381	0.043				0.0225	0.0003 to 0.0447	0.049			
Activity level	No sport	70	–	–	–	–	–	–						
	Sport	140	0.2442	0.0068 to 0.0421	0.036	0.0171	0.0024 to 0.0317	0.043						
	Athlete	210	0.0274	0.0096 to 0.0452	0.033	0.0322	0.0183 to 0.0020	0.022						
Physical Activity Questionnaire		420	−0.0397	−0.0552 to −0.0243	0.019							−0.0010	−0.0253 to 0.0234	0.701

*Age, gender and activity level as covariables.

†Age and cohort as covariables.

‡ Age, gender and Physical Activity Questionnaire as covariables.

BMI, body mass index.

10.1136/bjsports-2017-097626.supp9 Supplementary file 9

#### Activity levels and maximum cartilage alpha angle

**Figure 3:**
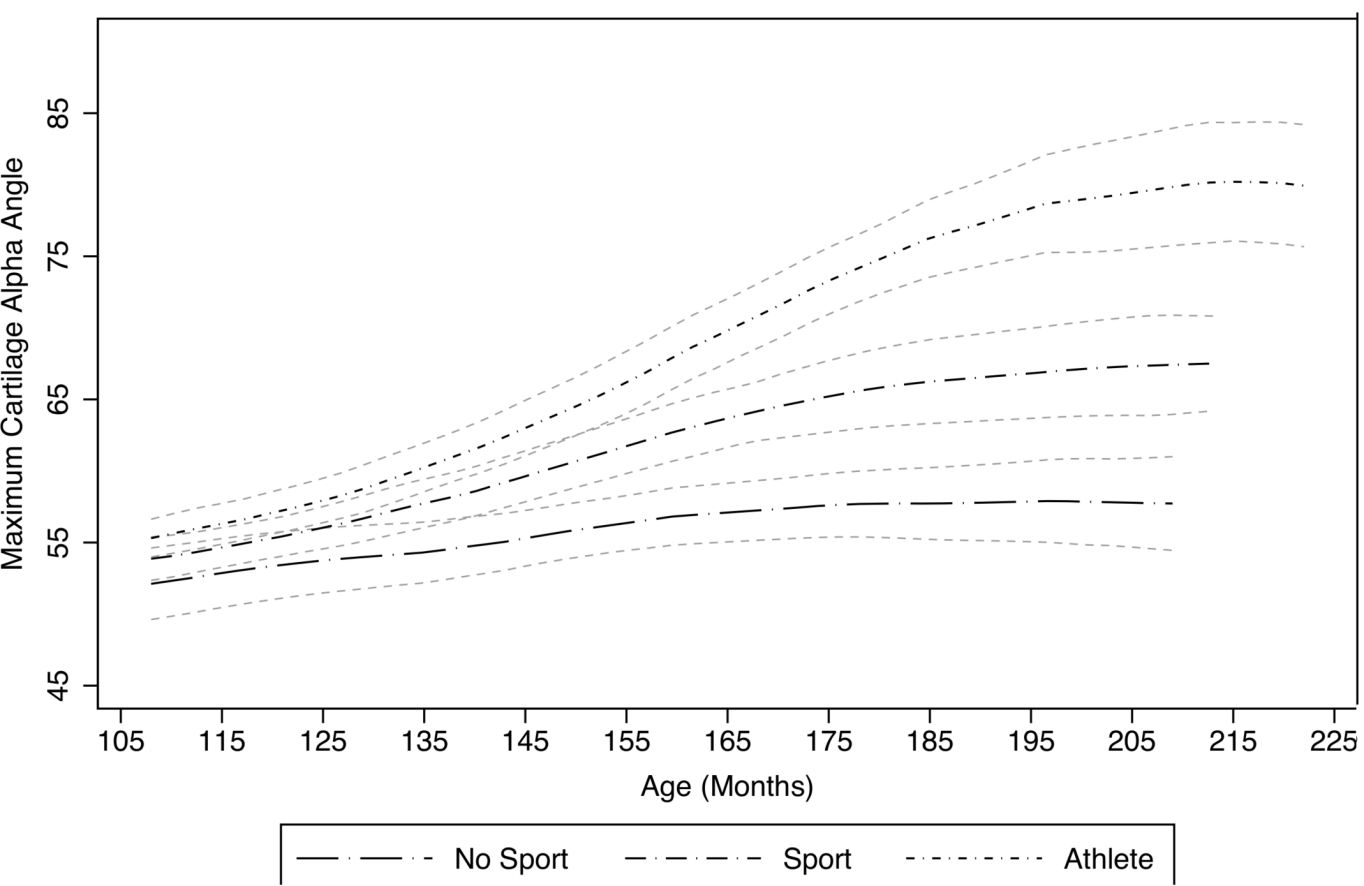
Maximum cartilage alpha angle with age for participants who play no regular sport (‘no sport’), play sport for their school or a club team (‘sport’) or compete at a national or international level (‘athlete’). Polynomial regression fit with 95% CI.

Maximum cartilage alpha angle increased with age and activity level (table 2 and figure 3).

Adjusting for age, female controls had a maximum cartilage alpha angle 7.4 degrees lower than male controls (p=0.004). Male controls had a maximum cartilage alpha angle 4.33 degrees lower than the SFC cohort (p=0.042).

Adjusting for age and gender, individuals who played sport for a school or club team had a maximum cartilage alpha angle 4.0 degrees greater than individuals who play no regular sport (p=0.041). Compared with individuals who play no regular sport, alpha angles were 7.6 degrees higher in individuals competing at a national or international level (p=0.035).

Adjusting for age and gender, maximum cartilage alpha angle increased with PAQ, but the relationship was not statistically significant. There was no statistically significant association between cartilage alpha angle and BMI or leg dominance when adjusting for age, gender, and activity levels.

### Physeal closure

In the SFC cohort, the physis was open in 135 hips and closed in 68 hips. Among male controls, the physis was open in 80 hips and closed in 24 hips. Among female controls, the physis was open in 32 hips and closed in 76 hips. The physis closed between ages 13 and 16 years in males and between 11 and 12 years in females. No difference was detected in the age of physeal closure between the SFC cohort and male controls.

#### Epiphyseal extension

Epiphyseal extension increased at each clockface position with age but was greatest at 11 o’clock prior to physeal closure and 1 o’clock after physeal closure (table 1). This limits the value of using maximal epiphyseal extension to investigate the pathogenesis of cam development, hence this study focused on factors associated with extension at 1 o’clock.

#### Activity levels and epiphyseal extension at 1 o’clock

Epiphyseal extension at 1 o’clock increased with age and activity level, as with cartilage alpha angle (table 2).

Adjusting for age, female controls had an epiphyseal extension measurement 0.006 lower than male controls (p=0.034), and male controls had an epiphyseal extension measurement 0.026 lower than the SFC cohort (p=0.049).

Adjusting for age and gender, individuals who play sport for a school or club team had an epiphyseal extension measurement 0.017 greater than individuals who play no regular sport (p=0.043). Compared with those who play no regular sport, epiphyseal extension was 0.032 greater in individuals competing at a national or international level (p=0.022).

Adjusting for age and gender, there was no statistically significant association between epiphyseal extension and PAQ. There was no statistically significant association between epiphyseal extension and leg dominance when adjusting for age, gender, and activity level, although BMI nearly reached statistical significance (p=0.051).

#### Physeal tilt

With increasing age, physeal tilt decreased at 11 o’clock and 12 o’clock (extension of inferior epiphysis relative to superior epiphysis) and increased at 2 o’clock and 3 o’clock (extension of anterior epiphysis relative to posterior epiphysis) (table 1). After physeal closure, physeal tilt was greatest at 1 o’clock and in the SFC cohort.

### Relationship between epiphyseal measurements and alpha angle

The strongest correlation between epiphyseal extension and cartilage alpha angle was at 1 o’clock, with progressively weaker correlations either side of this location. This relationship was only seen in male participants. In the SFC cohort, 50% of the variability in cartilage alpha angle was explained by adjacent epiphyseal extension (p=0.043) (see online supplementary table S2).

The relationship between physeal tilt and cartilage alpha angle was limited. A positive correlation at 3 o’clock only reached statistical significance in the SFC cohort (p=0.014). This positive correlation indicates anterior relative to posterior epiphyseal extension with increasing alpha angles (opposite to that expected in slipped upper femoral epiphysis (SUFE) (see online supplementary table S2).

### Qualitative MRI morphology

**Figure 4:**
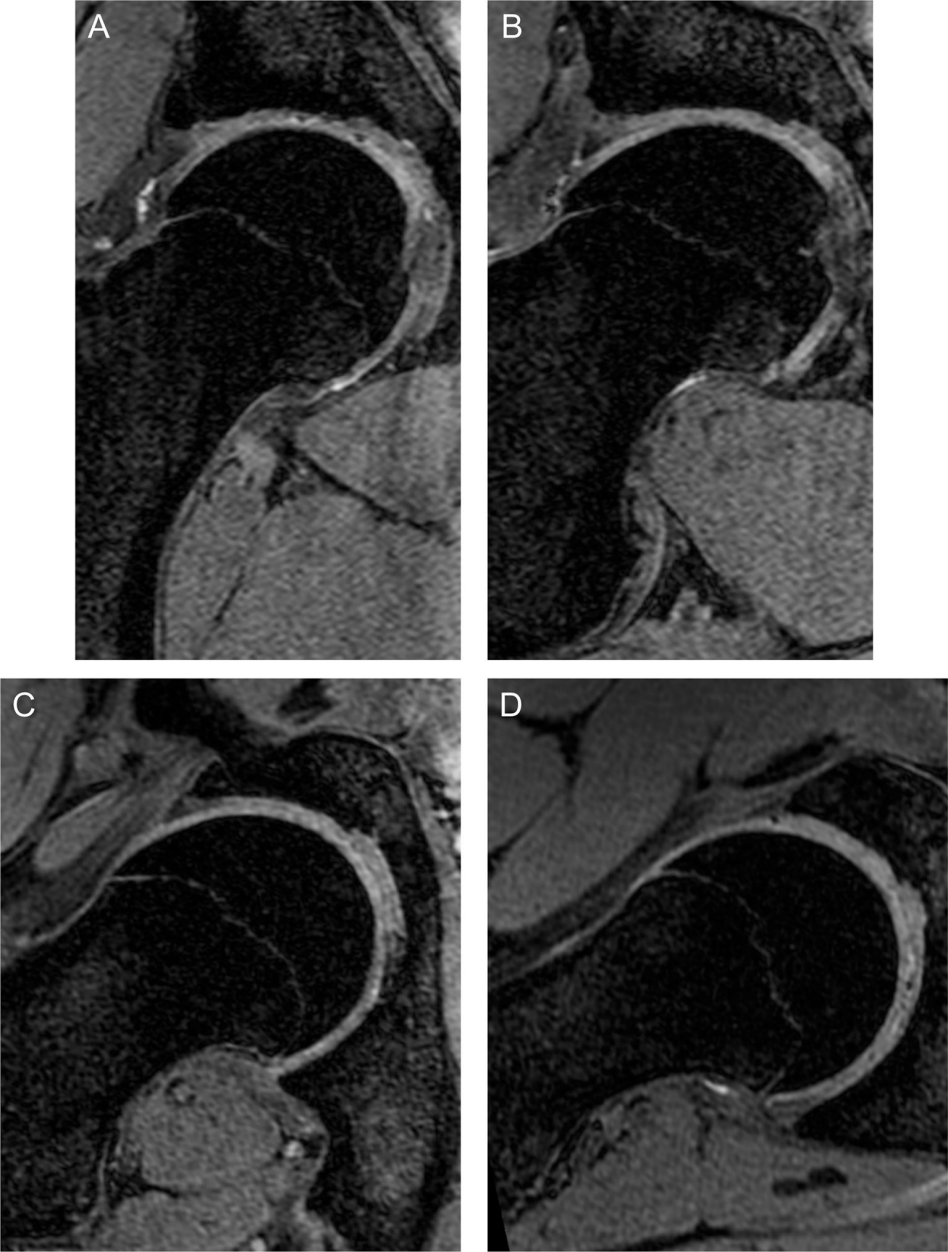
Three-dimensional water selective fluid (WATSf) MRI of SFC player aged 16 years with pronounced cam morphology. Epiphyseal hypertrophy and extension is maximal at 1 o’clock but also present in adjacent positions: 12 o’clock (**A**), 1 o’clock (**B**), 2 o’clock (**C**) and 3 o’clock (**D**).

Epiphyseal hypertrophy and extension was the most frequently observed source of cam morphology among study participants and was particularly prevalent among the SFC cohort beyond 14 years of age. Although usually most pronounced at 1 o’clock, this hypertrophy and extension was concurrently observed at adjacent positions (figure 4). No epiphyseal extension was observed in participants aged 9 years, however, by 11 years of age there was often early extension that may progress to cam morphology (see online supplementary figure S4). Epiphyseal extension was rarely observed at 3 o’clock and 11 o’clock. Cam morphology at 3 o’clock was more frequently dictated by femoral neck retroversion and appeared independent of anterosuperior epiphyseal extension (see online supplementary figure S5). Cam morphology at 11 o’clock and 12 o’clock was frequently secondary to non-osseous tissue at the head-neck junction (see online supplementary figure S6).

10.1136/bjsports-2017-097626.supp6 Supplementary file 6

10.1136/bjsports-2017-097626.supp7 Supplementary file 7

10.1136/bjsports-2017-097626.supp8 Supplementary file 8

## Discussion

This study demonstrates that intense sporting activity during youth is associated with the development of cam morphology arising secondary to epiphyseal hypertrophy and extension along the anterosuperior femoral neck.

### Age of cam development

Cam morphology was quantified using the alpha angle. The magnitude of alpha angle that represents pathological morphology is likely to depend on femoral morphology, acetabular morphology, cartilage susceptibility and activity levels.^19^ Alpha angles were uniform across all participants aged 9 years; however, with increasing age there was an increasing range of alpha angle values consistent with cam development in a proportion of individuals. Cartilage alpha angles increased as early as age 10 years and qualitatively this represented soft-tissue hypertrophy at the head-neck junction that we propose precedes extension of the ossified epiphysis. Bone alpha angles were never elevated secondary to cam morphology before age 12 years, as described in similar studies.^3 5^ This observation suggests that measurements confined to osseous morphology are insensitive to early cam development and explains why loss of internal rotation precedes radiographic cam morphology (see online supplementary figure S7).^7^

The greatest increase in cartilage alpha angle occurred between age 12 and 14 years, with no statistically significant increase beyond age 14 years. This suggests cam development precedes physeal closure, as concluded in comparable studies.^3 5 7^ There are studies that report higher alpha angles when the physis is open. We reproduced this observation when measuring bone rather than cartilage alpha angles at 2 o’clock and 3 o’clock. In the skeletally immature hip, the ossified femoral head is small relative to the metaphysis, giving rise to raised bone alpha angles that do not necessarily reflect cam morphology (see online supplementary figure S8). Bone alpha angle measurements must be interpreted with caution in the immature skeleton.

10.1136/bjsports-2017-097626.supp10 Supplementary file 10

Prevalence of cam morphology in females was insufficient to allow robust comparisons in age of cam development between sexes. Crucially, interventions to reduce the risk of cam development should commence before age 10 years and may be ineffective beyond skeletal maturity.

### Pathogenesis

Epiphyseal hypertrophy and extension represented the salient mechanism of cam development in this cohort. The greatest increase and absolute values of cartilage alpha angle and epiphyseal extension were at the 1 o’clock position, as in comparable studies.^13^ We demonstrate stronger correlations between epiphyseal extension and alpha angle than previously reported.^15^ In males, 50% of the variability in cartilage alpha angle was explained by adjacent epiphyseal extension. This supports the proposal that epiphyseal extension is a salient mechanism of cam development, but not the sole mechanism. Importantly, our adopted measure of epiphyseal extension provides only limited assessment of epiphyseal morphology and is not sensitive to epiphyseal hypertrophy without extension that also gives rise to cam morphology (see online supplementary figure S9). Active shape modelling is likely to provide improved assessment of epiphyseal morphology. The relationship between epiphyseal extension and alpha angle is particularly weak in females where alternative cam development pathogenesis may dominate, such as asphericity secondary to dysplasia.

10.1136/bjsports-2017-097626.supp11 Supplementary file 11

Cam morphology at 3 o’clock appeared more frequently dictated by femoral neck retroversion than epiphyseal morphology; however, epiphysiel extension was responsible for the highest alpha angles. Retroversion was not formally quantified, but unlike epiphyseal extension, was evident among all age groups and we speculate a different pathogenesis. Interestingly, 3 o’clock was the only position where epiphyseal tilt correlated with cartilage alpha angle, compatible with anterior epiphyseal extension as the cause of cam morphology. Our proposed explanation for the absence of correlation at superior clockface positions is variation in physis orientation in the coronal plane that is not seen in the axial plane. There was no qualitative or quantitative evidence of SUFE; however, epiphyseal changes may occur in response to stress across the physis without translation. Forces are greater with a more vertically orientated physis, which gives higher inferior epiphysiel extension values and counters increasing epiphyseal tilt measurements secondary to superior epiphyseal extension. Inferior epiphyseal extension was not associated with activity levels.

### Associations with cam morphology

Cartilage alpha angle and epiphyseal extension were greater in males than females, as widely reported in general population cohorts.^22^ This gender difference remains unexplained. Interestingly, the sex ratio for cam morphology is similar to SUFE,^23^ and perhaps the physis is more responsive to load in males. BMI was not associated with cartilage alpha angle or epiphyseal extension.

Activity levels were strongly associated with cartilage alpha angle and epiphyseal extension. There was a stepwise increase from participants who play no regular sport, to participants who play sport for a school or club, and then participants who compete at a national or international level. Some studies also found increased alpha angles^4^ and epiphyseal extension^15^ in athletes compared with controls, but others found no statistically significant differences, potentially due to insufficient statistical power.^12 24^ Our large cohort provides strong evidence that high activity levels during adolescence promote cam development secondary to epiphyseal extension with a dose-response relationship. This finding validates the proposed dose-response relationship between activity levels and cam morphology in other studies.^3 25^

Activity levels are challenging to quantify. In this study, competitive level of sport participation was used as a surrogate measure of cumulative exercise intensity. General activity levels measured using the PAQ were not associated with cam morphology. PAQ is particularly responsive to exercise frequency and the limitation of this self-reported score for assessing sporting activity is illustrated by the significant decrease in PAQ with age among all study groups. Mean PAQ within the SFC cohort was 3.44 (SD 0.43) age 9–10 years, 3.36 (SD 0.68) age 11–12 years, 2.98 (SD 0.74) age 13–14 years, 2.51 (SD 0.66) age 15–16 years and 2.17 (SD 0.42) age 17–18 years. However, training load for academy football players increases with age from approximately 5 to 8 hours each week for under 9–12 years, 8–12 hours each week for under 13–16 years, to greater than 12 hours each week at under 18–23 years. Electronic activity trackers may aid future studies.

There was no difference in cartilage alpha angle or epiphyseal extension between dominant and non-dominant legs, reproducing findings of a previous study.^12^ This observation counters hypotheses that kicking is responsible for cam formation by imparting forces across the physis that stimulate epiphyseal extension. Footballers are encouraged to kick with both feet, which may partially explain the finding; however, no difference was detected between control individuals who play kicking and non-kicking sports. Repetitive high-intensity hip loading during sport played throughout a critical period of hip development may be the salient risk factor for cam development. Further research is required to determine whether similar adaptation to activity takes place within the acetabulum.

### Limitations

Study limitations include the demonstration of association rather than causality from this cross-sectional data. In addition, it is possible that the control group is not entirely representative of the general population as individuals who regularly participate in sport may be more likely to volunteer for projects investigating effects of exercise. Nevertheless, there were statistically significant differences in hip morphology between the control and athlete cohorts. Alpha angle has a low positive predictive value for future pain and osteoarthritis, hence the clinical relevance of our findings remains unclear, but will be addressed with the longitudinal element of the study.

## Conclusions

Intense sporting activity during adolescence is associated with cam morphology secondary to epiphyseal hypertrophy and extension along the anterosuperior femoral neck. Cam morphology is first evident as cartilaginous hypertrophy age 10 years. The pathogenesis of cam development currently remains insufficiently understood to recommend activity modification. The known cardiovascular and health benefits of exercise outweigh potentially adverse effects on hip development. However, males participating in high-level sports during adolescence are at particularly elevated risk of developing cam morphology and secondary hip pathology.

**What are the findings?**

- Osseous cam morphology is preceded by cartilaginous hypertrophy at the femoral head-neck junction. This appearance is first evident at 10 years of age.
- Sporting activity during adolescence is strongly associated with the development of cam morphology with a dose-response relationship. The salient mechanism is epiphyseal hypertrophy and extension along the anterosuperior femoral neck.
- Cam morphology is significantly more prevalent in males. General activity levels, leg dominance and kicking sports are not independently associated with cam morphology.
- Males participating in competitive sport during adolescence are at particularly elevated risk of developing cam morphology.

**How might it impact on clinical practice in the future?**

- A history of competitive sport during adolescence is strongly associated with cam morphology. Clinicians should be alert to the risk of secondary hip pathology.
- MRI is recommended to diagnose cam morphology in skeletally immature individuals as early non-osseous cam morphology may not be evident on radiographs.
- Proposed interventions to prevent the development of cam morphology should commence prior to 10 years of age.
