# Supplementary figures and images for: Physical activity during adolescence and the development of cam morphology: a cross-sectional cohort study of 210 individuals
Source: Br J Sports Med. 2017 Aug 10;52(9):601–10. doi: 10.1136/bjsports-2017-097626 (PMC5909766; doi:10.1136/bjsports-2017-097626)

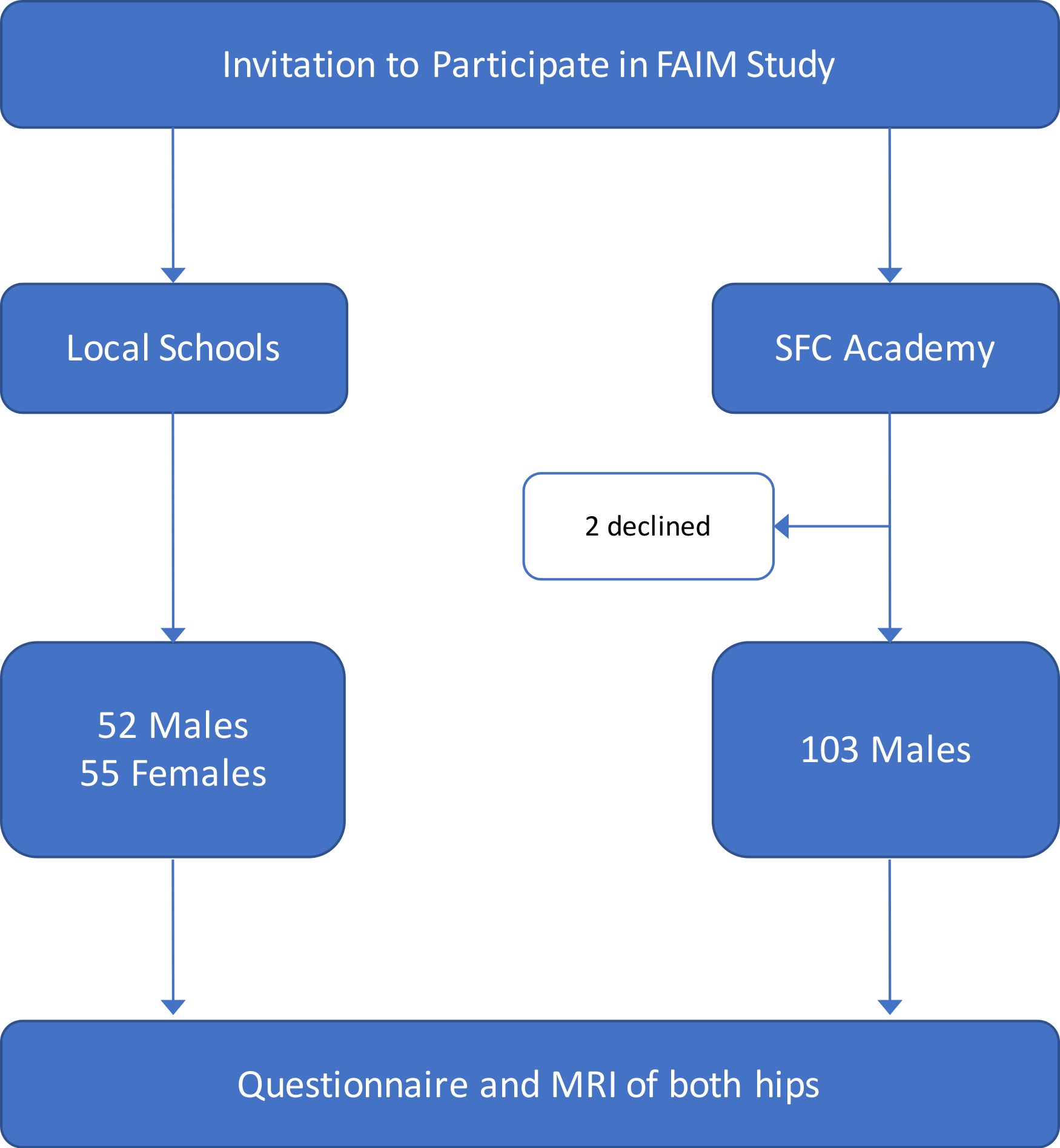

Supplement: Supplementary file 1 [file bjsports-2017-097626supp001.jpg]

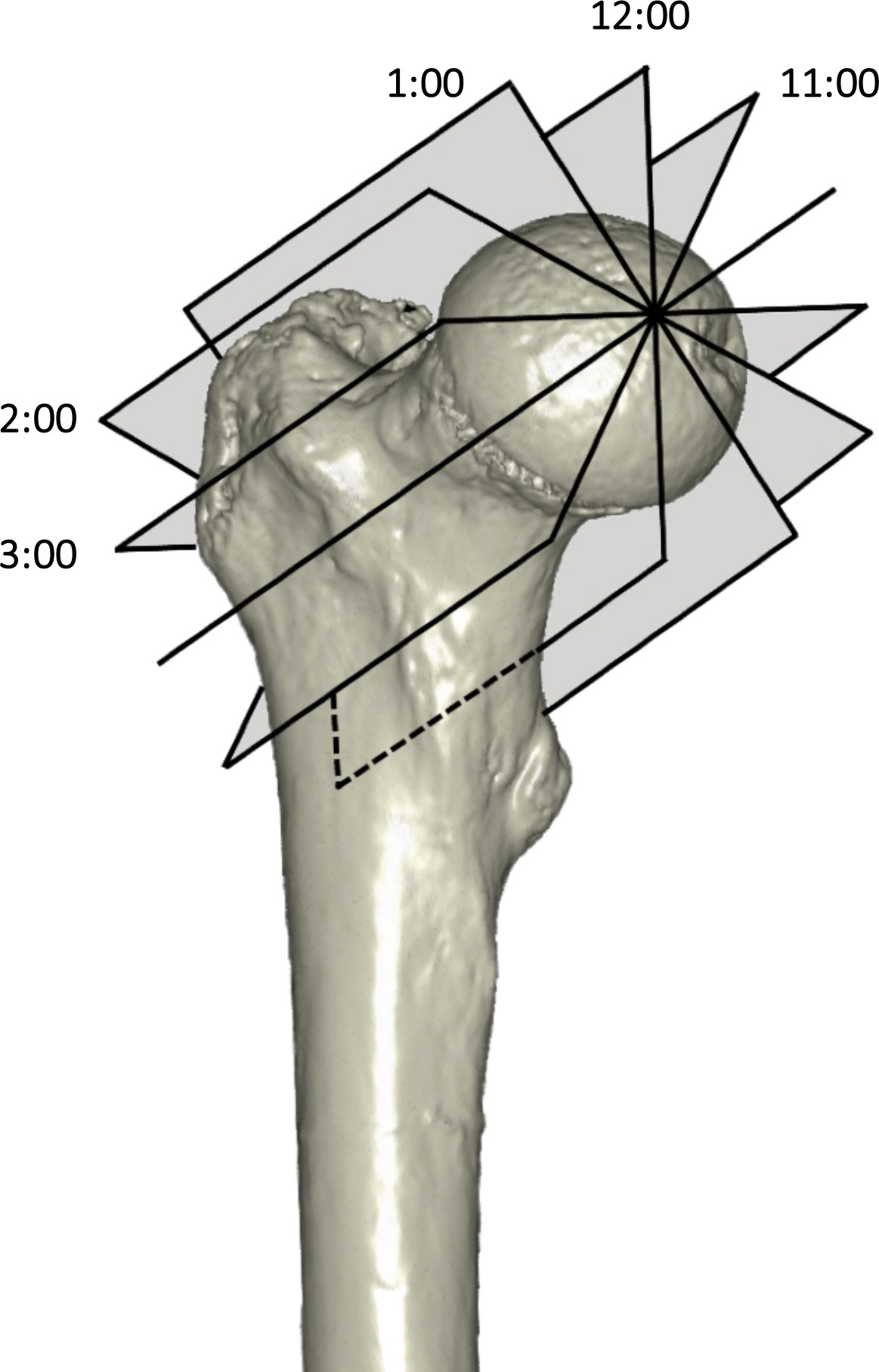

Supplement: Supplementary file 4 [file bjsports-2017-097626supp004.jpg]

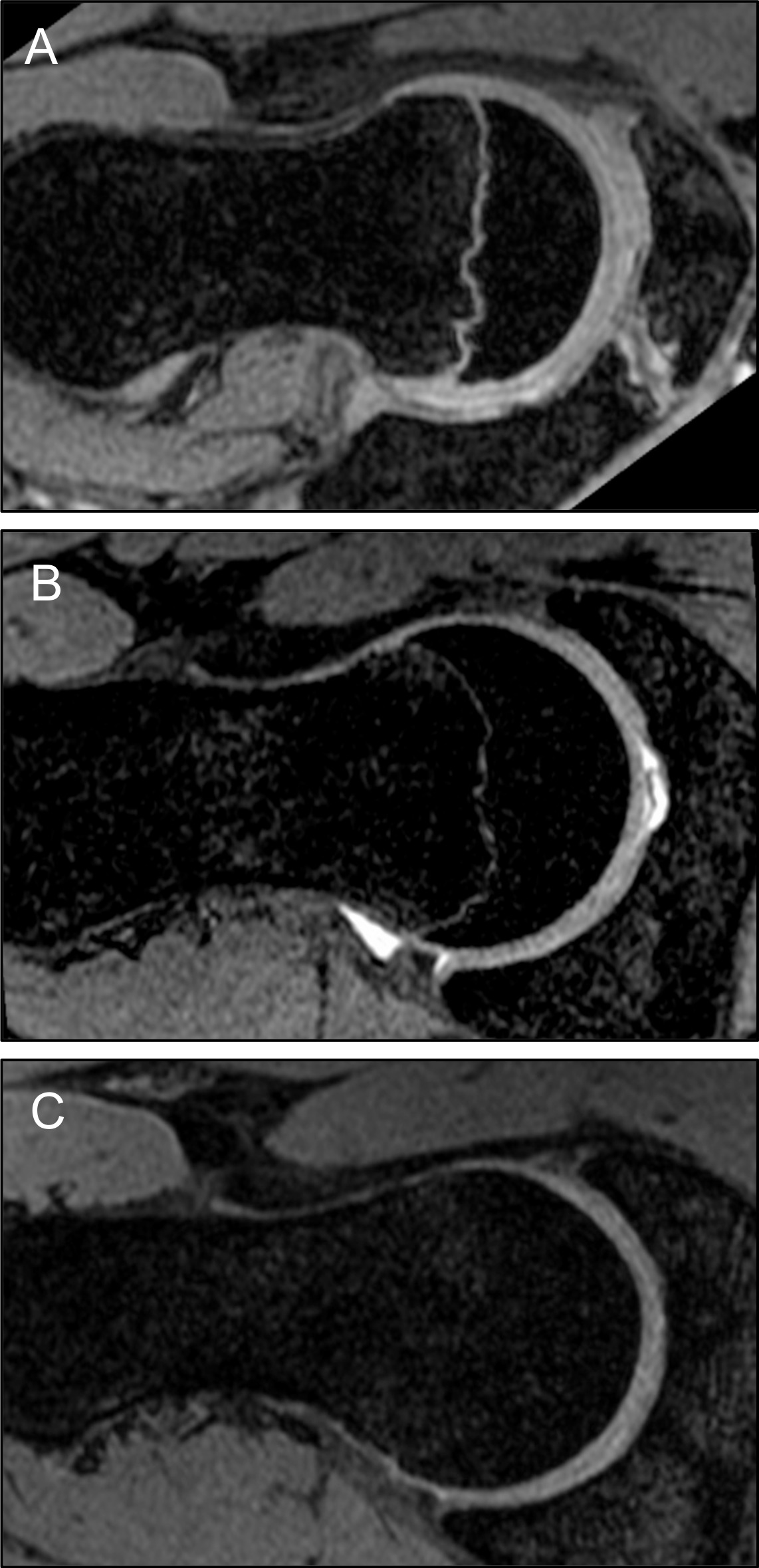

Supplement: Supplementary file 5 [file bjsports-2017-097626supp005.jpg]

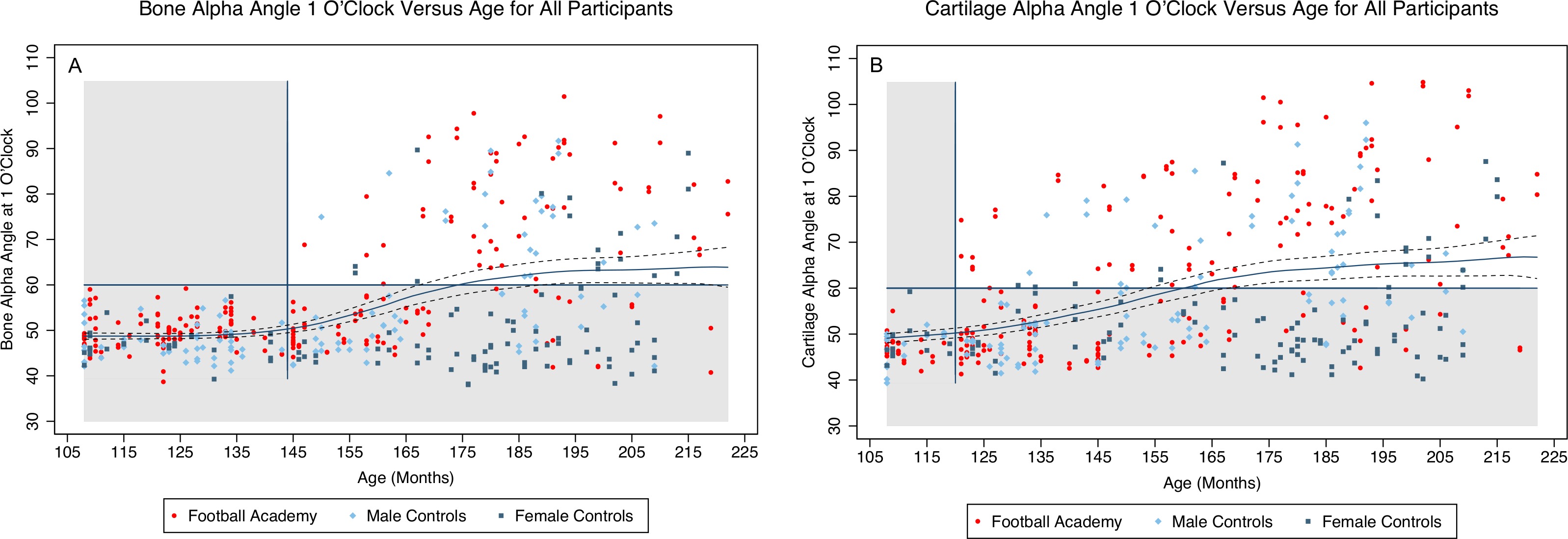

Supplement: Supplementary file 9 [file bjsports-2017-097626supp009.jpg]

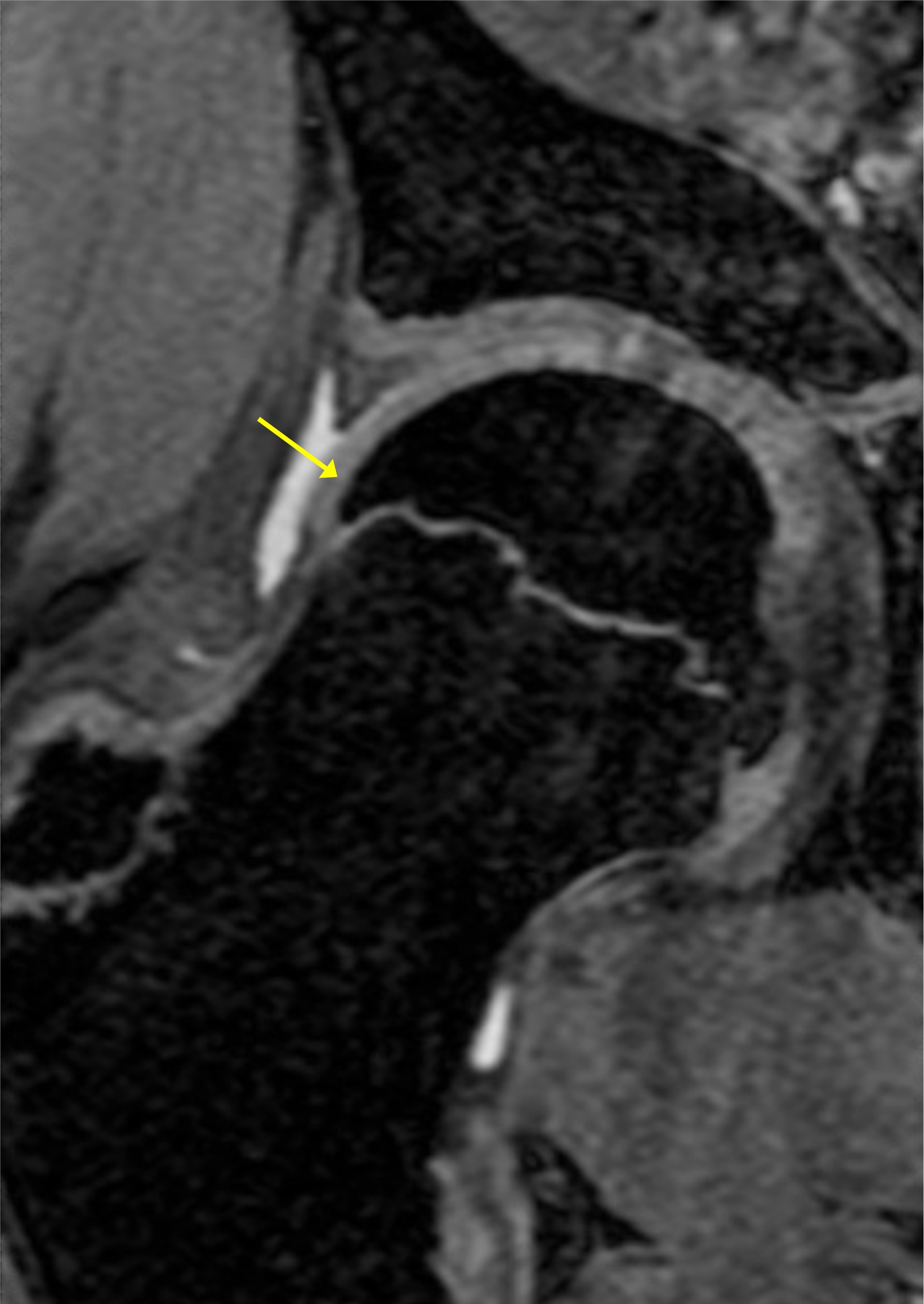

Supplement: Supplementary file 6 [file bjsports-2017-097626supp006.jpg]

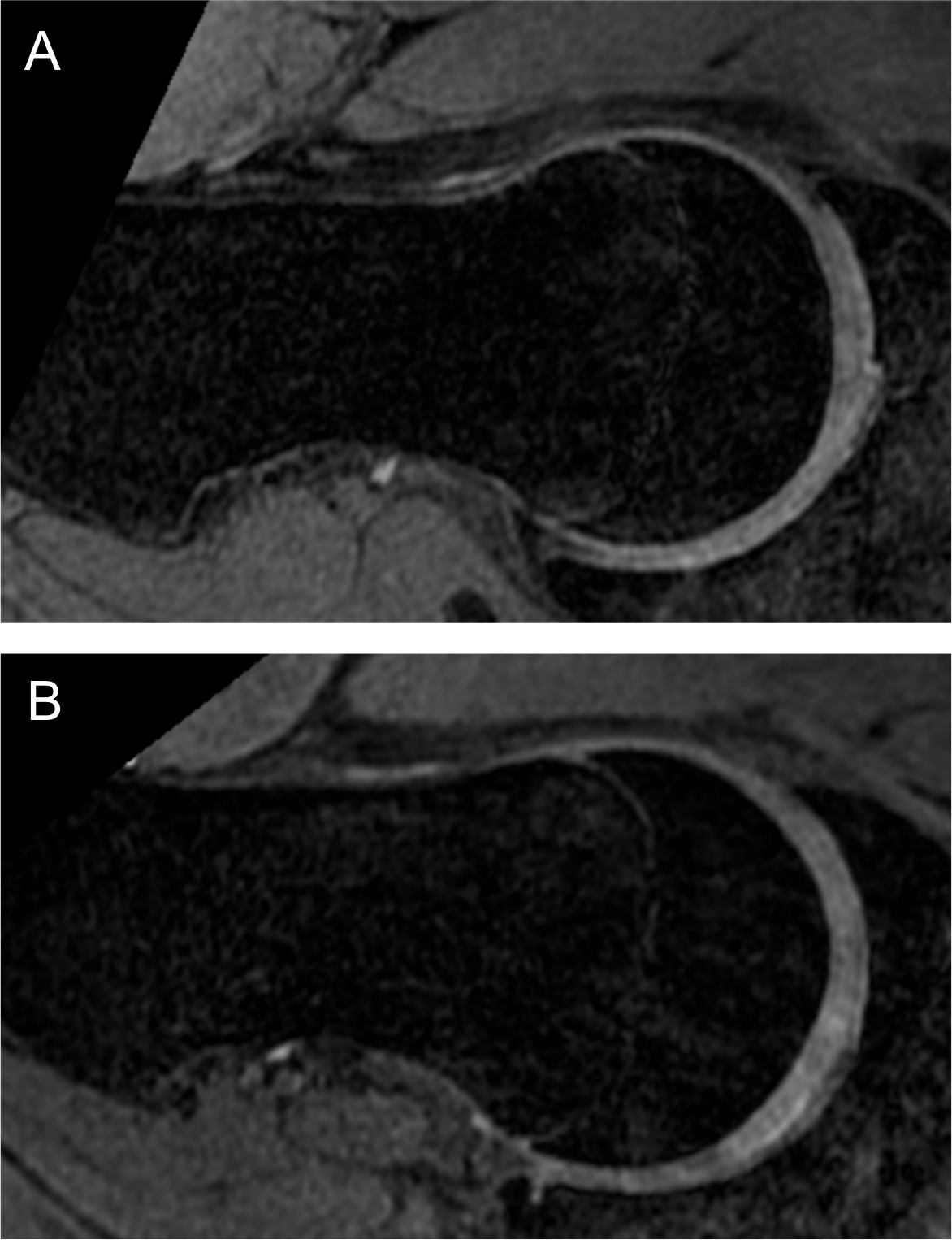

Supplement: Supplementary file 7 [file bjsports-2017-097626supp007.jpg]

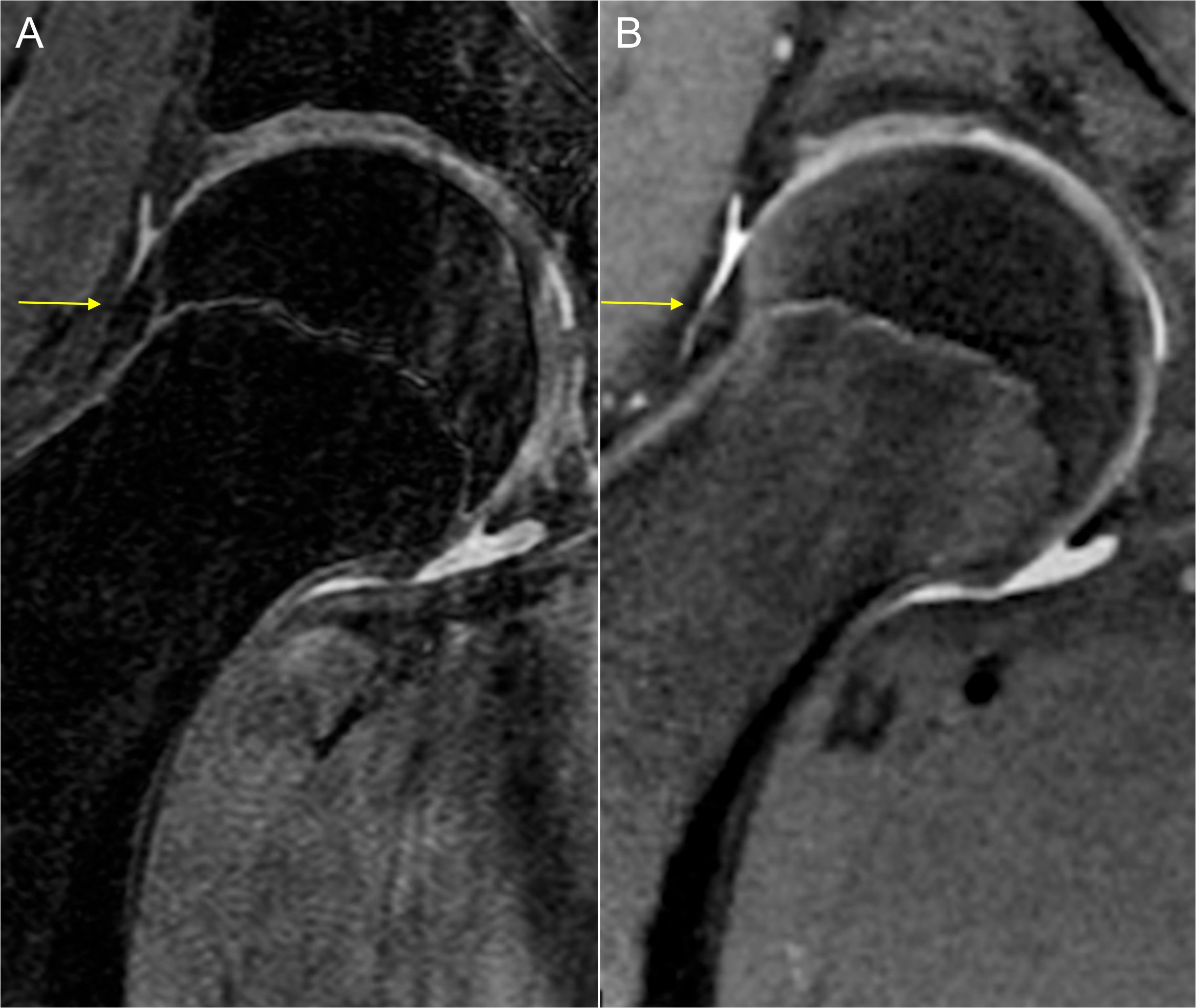

Supplement: Supplementary file 8 [file bjsports-2017-097626supp008.jpg]

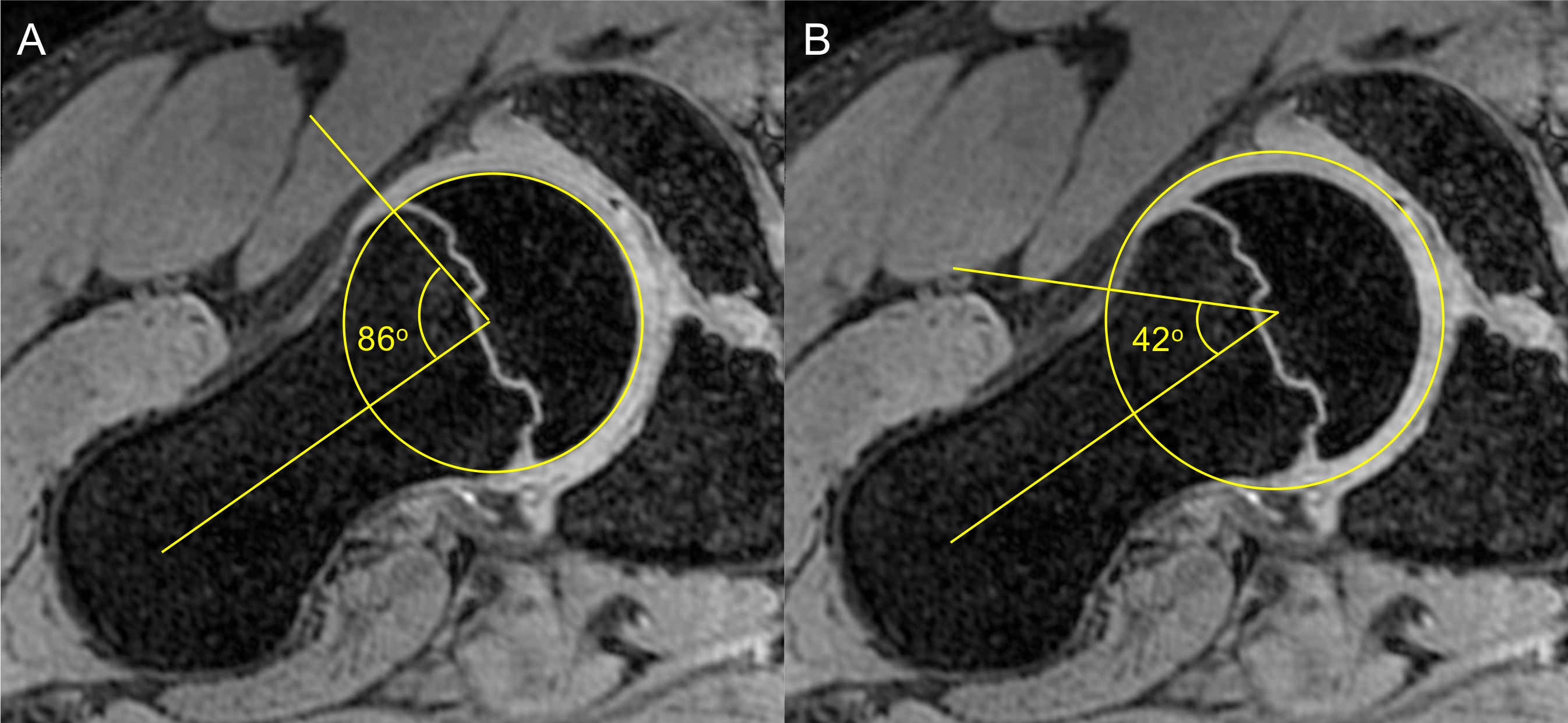

Supplement: Supplementary file 10 [file bjsports-2017-097626supp010.jpg]

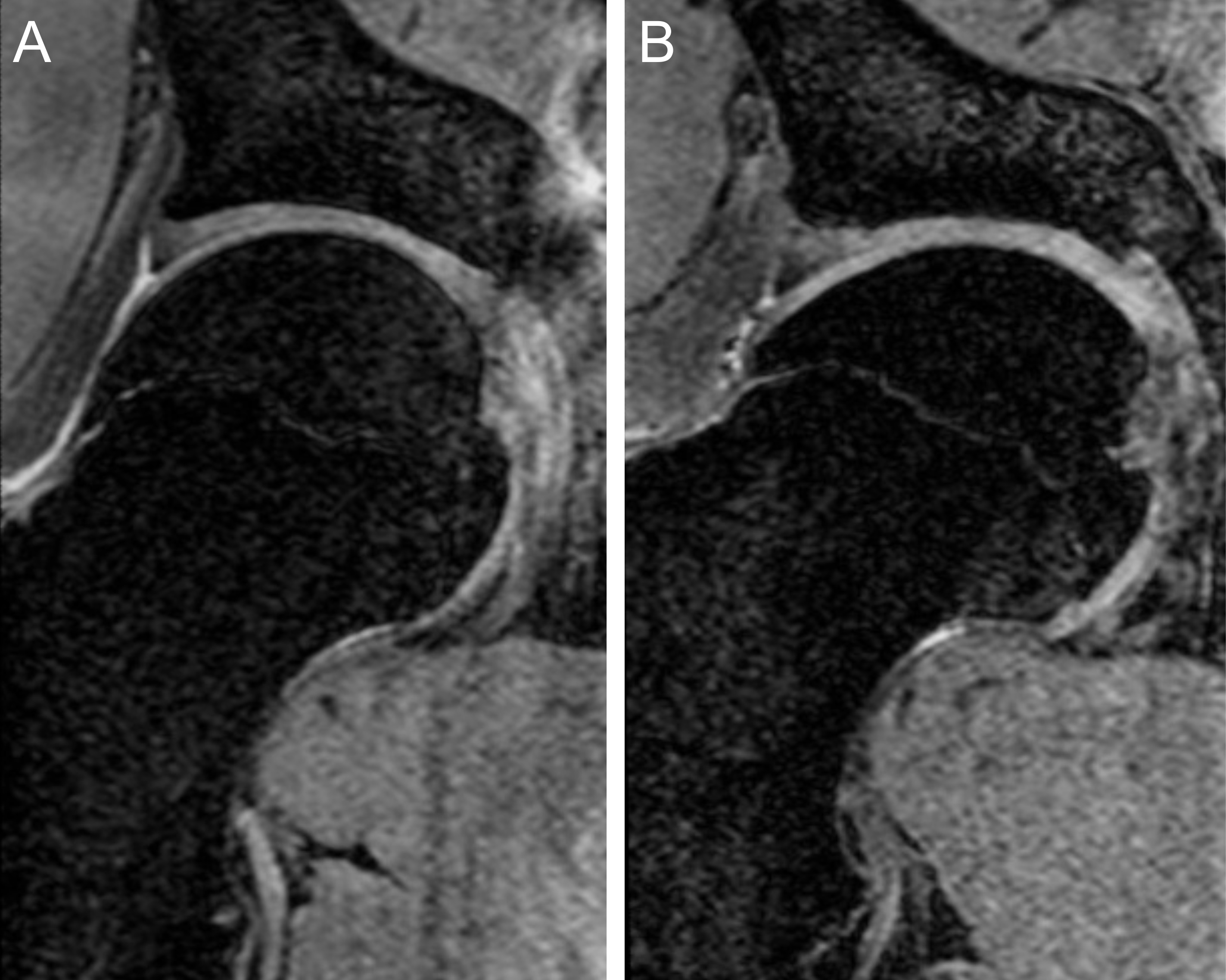

Supplement: Supplementary file 11 [file bjsports-2017-097626supp011.jpg]
