# Supplementary material for: Physical activity during adolescence and the development of cam morphology: a cross-sectional cohort study of 210 individuals
Source: Br J Sports Med. 2017 Aug 10;52(9):601–10. doi: 10.1136/bjsports-2017-097626 (PMC5909766; doi:10.1136/bjsports-2017-097626)
Supplement: Supplementary file 2 [file bjsports-2017-097626supp002.docx]

**SUPPLEMENTARY DATA:**

Power Calculation:

Sample size calculations were based on existing literature at 80% power and 5% significance using a two-sided t-test. Carsen et al. reported a mean change in alpha angle of 4.85 degrees (SD 7.05) between individuals with an open and closed physis.^3^ We calculated that a minimum of 35 individuals were required either side of physeal closure to study morphological changes during adolescence. Siebenrock et al. reported a mean difference in alpha angle between athletes and controls of 6.2 degrees (SD 7.4) in individuals with an open physis and 16.9 degrees (SD 7.6) with a closed physis.^4^ We calculated that a minimum of 24 individuals pre-physeal closure and 5 individuals post-physeal closure were required in the male control group for comparisons with male athletes. A 1:1 recruitment strategy was adopted for male and female controls.

MRI Protocols:

1. 3D Water Selective Fluid (WATSf): Repetition time (TR) 13.65ms, echo time (TE) 6.9ms, flip angle 30 degrees, bandwidth 145Hz/pixel, interpolated voxel size 0.29mm x 0.29mm x 0.4mm, averages 2, acquired in true sagittal orientation.
2. 3D Proton Density Fat Saturation (PDFS): Repetition time (TR) 1300ms, echo time (TE) 40ms, flip angle 90 degrees, bandwidth 243Hz/pixel, interpolated voxel size 0.625mm x 0.625mm x 0.65mm, averages 2, acquired in true sagittal orientation.

Reproducibility Data:

The intra-observer ICC for alpha angle measured on MRI radial planes (five planes in 20 hips: ten with open physis and ten with closed physis) was 0.962 (95% CI 0.931 – 0.977) and the inter-observer ICC 0.956 (95% CI 0.935 – 0.970).

The intra-observer ICC for epiphyseal extension measured on MRI radial planes (five planes in 20 hips: ten with open physis and ten with closed physis) was 0.976 (95% CI 0.964 – 0.984) and the inter-observer ICC 0.970 (95% CI 0.947 – 0.982).

**SUPPLEMENTARY FIGURE LEGENDS:**

Supplementary Figure 1:

Flowchart outlining recruitment of the study cohort.

Supplementary Figure 2:

Schematic representation of radial images around the axis of the femoral neck. The coronal plane (12 o’clock) was positioned parallel to the axis of the proximal femur diaphysis. The 3 o’clock position was anterior for both left and right hips.

Supplementary Figure 3:

The physis of each hip was scored as either ‘open’ (A), ‘partially closed’ (B), or ‘closed’ (C). A physis was denoted partially closed when there was contact between the epiphysis and metaphysis but the physis remained visible on a WATSf MRI sequence. Physeal closure was treated as a binary variable and a partially closed physis was considered closed to enhance classification reproducibility.

Supplementary Figure 4:

WATSf MRI of 11 year old SFC player with evidence of early epiphyseal extension (arrow) at the 1 o’clock position.

Supplementary Figure 5:

WATSf MRI at 3 o’clock position in SFC players at the time of physeal closure. Preservation of the metaphyseal concavity (A) results in a spherical femoral head-neck configuration, however, retroversion of the femoral neck and loss of the metaphyseal concavity (B) gives rise to cam morphology. This retroversion was independent of epiphyseal hypertrophy and extension. Epiphyseal tilt measurements are the same for both players.

Supplementary Figure 6:

WATSf (A) and PDFS (B) MRI of the femoral head-neck junction at the 12 o’clock position in a 13 year old academy football player. Discrete fibrous tissue that may represent the perichondrial fibrous ring of La Croix obliterates the concavity. It is not known whether this finding persists beyond adolescence.

Supplementary Figure 7:

WATSf MRI illustrating bone (A) and cartilage (B) alpha angle measurements at the 3 o’clock in a 9 year old male. In early adolescence age the ossified femoral head remains small relative to the metaphysis giving artificially elevated bone alpha angles.

Supplementary Figure 8:

Comparison between bone (A) and cartilage (B) alpha angle versus age in all participants at the 1 o’clock position. A proposed diagnostic threshold for cam morphology is 60 degrees and is illustrated with a horizontal line.^20^ Elevated cartilage alpha angles are first evident age 10 years (B) whereas bone alpha angles are not elevated until age 12 years (A), illustrated with vertical lines. Bone alpha angle measurements are insensitive to early cam morphology.

Supplementary Figure 9:

WATSf MRI of 14 year old SFC players at 1 o’clock position. Epiphyseal extension (A) is associated with the development of cam morphology, however, cam morphology may also result from epiphyseal hypertrophy without pronounced extension (B).
