# Supplementary material for: Physical activity during adolescence and the development of cam morphology: a cross-sectional cohort study of 210 individuals
Source: Br J Sports Med. 2017 Aug 10;52(9):601–10. doi: 10.1136/bjsports-2017-097626 (PMC5909766; doi:10.1136/bjsports-2017-097626)
Supplement: Supplementary file 3 [file bjsports-2017-097626supp003.docx]

Supplementary Table 1: Study Participants: Southampton Football Club (SFC) and Controls.

|  | **Participant Numbers** | | |
| --- | --- | --- | --- |
| **Age** | SFC | Controls | |
| (years) | Male | Male | Female |
| 9 | 16 | 11 | 9 |
| 10 | 17 | 8 | 4 |
| 11 | 13 | 7 | 3 |
| 12 | 12 | 7 | 3 |
| 13 | 11 | 6 | 5 |
| 14 | 9 | 2 | 6 |
| 15 | 11 | 10 | 9 |
| 16-18 | 14 | 5 | 16 |
| Total | 103 | 52 | 55 |

Supplementary Table 2: Regression Coefficients for Epiphyseal Extension and Epiphyseal Tilt with Alpha Angle in All Age Groups

| Regression Coefficients for Epiphyseal Extension versus Alpha Angle | | | | | | | | |
| --- | --- | --- | --- | --- | --- | --- | --- | --- |
| Clockface Position | Football Academy | | Male Controls | | Female Controls | | All Participants | |
|  | Regression Coefficient | R^2^ value | Regression Coefficient | R^2^ value | Regression Coefficient | R^2^ value | Regression Coefficient | R^2^ value |
| 11 O’Clock | 99.81 | 0.229 | 47.15 | 0.082 | 36.08 | 0.030 | 80.12 | 0.154 |
| 12 O’Clock | 116.43 | 0.401 | 101.23 | 0.279 | 55.86 | 0.130 | 99.21 | 0.299 |
| 1 O’Clock | 126.24 | 0.498 | 130.78 | 0.491 | 53.62 | 0.129 | 112.99 | 0.398 |
| 2 O’Clock | 108.66 | 0.463 | 82.67 | 0.423 | 38.88 | 0.163 | 79.89 | 0.332 |
| 3 O’Clock | 87.94 | 0.330 | 68.48 | 0.296 | 22.61 | 0.054 | 59.40 | 0.202 |
|  | | | | | | | | |
| Regression Coefficients for Epiphyseal Tilt versus Alpha Angle | | | | | | | | |
| Clockface Position | Football Academy | | Male Controls | | Female Controls | | All Participants | |
|  | Regression Coefficient | R^2^ value | Regression Coefficient | R^2^ value | Regression Coefficient | R^2^ value | Regression Coefficient | R^2^ value |
| 11 O’Clock | -12.98 | 0.039 | -9.90 | 0.077 | -1.99 | 0.001 | -5.641 | 0.011 |
| 12 O’Clock | -2.01 | 0.001 | -13.62 | 0.028 | -5.73 | 0.010 | 0.065 | 0.000 |
| 1 O’Clock | 8.41 | 0.043 | 6.80 | 0.008 | 1.33 | 0.001 | 8.286 | 0.015 |
| 2 O’Clock | 12.55 | 0.048 | 12.68 | 0.038 | 1.65 | 0.002 | 9.540 | 0.028 |
| 3 O’Clock | 49.32 | 0.270 | 27.77 | 0.103 | 6.12 | 0.014 | 30.486 | 0.134 |
